# Urolastic—A New Bulking Agent for the Treatment of Women with Stress Urinary Incontinence: Outcome of 12 Months Follow Up

**DOI:** 10.1155/2013/724082

**Published:** 2013-12-22

**Authors:** Janusz Zajda, Fawzy Farag

**Affiliations:** ^1^MOCONTI Sp.z o.o Urological Office, Warszawa, Poland; ^2^Department of Urology, IATROS NZOZ, Warszawa, Poland; ^3^Department of Urology, Radboud UMC, P.O. Box 9101, 6500 HB Nijmegen, The Netherlands; ^4^Department of Urology, Sohag University Hospital, Egypt

## Abstract

*Objective*. To evaluate the efficacy and safety of the new injectable implant, Urolastic, in women with stress urinary incontinence (SUI) after 12-month followup. *Materials and Methods*. A prospective, cohort study included adult women with SUI. Patients were treated with Urolastic periurethral injections under local anaesthesia. The injection procedure was repeated after 6 weeks when indicated. Patients were evaluated for efficacy and safety parameters 6 weeks, 3 months, and 12 months after therapy. *Results*. Twenty women 56 (33–71) years old were included. Thirteen patients (65%) received one injection each (overall average of 2,1 mL); 7 patients (35%) received a second injection. Nineteen patients complete the 12-month followup. The mean Stamey incontinence grade significantly decreased from 1.9 at baseline to 0.4 at 12 months (visit IV) (*P* < 0.001). None of the patients were dry at baseline; 68% of them were dry at 12 months. The mean number of incontinence episodes significantly decreased from 6/day at baseline to 1.6/day at visit IV (*P* < 0.001). Reduction in pad weight went from 20.2 to 7.8 g at one year. The mean I-QoL score significantly increased from 51 at baseline to 76 at visit IV (*P* < 0.001). Six patients (30%) developed minor complications related to the injection procedure. *Conclusions.* Urolastic is effective and long-standing urethral bulking agent with moderate adverse events.

## 1. Introduction

Urinary incontinence (UI) is defined as “the complaint of any involuntary leakage of urine.” It is classified as stress urinary incontinence (SUI) when the leakage occurs on efforts or exertion, or on sneezing or coughing; urge urinary incontinence (UUI) when leakage is accompanied by or immediately preceded by urgency; or mixed urinary incontinence (MUI) when leakage is associated with urgency and also with efforts or exertion, or when sneezing or coughing [1].

UI is common in women with negative impact on their life quality. Hunskaar et al. [2] reported about 35% prevalence of UI in a study that included women from 4 European countries. SUI was the most overall prevalent subtype of UI in these women.

Surgical approach could provide ultimate cure for the SUI. However, a substantial number of women with SUI seek for less invasive procedures with lower risk of complications [3].

Injection therapy with urethral bulking agents is a good example for a less invasive treatment of the SUI. Many urethral bulking materials have been used in clinical trials. An ideal bulking agent should be biocompatible, nonimmunogenic, causes no fibrosis after infiltration of the urethral tissue, nonantigenic, and acellular [4]. To date, none of these agents proved to be better than any other agents [5].

Urolastic (Urogyn BV, Nijmegen, The Netherlands) is a new bulking agent that consists of vinyldimethyl terminated polydimethylsiloxane (PDMS) polymer, tetrapropoxysilane cross-linking agent, platinum vinyltetramethyl siloxane complex catalyst, and titanium dioxide radiopacifying agent.

The aim of the current study was to determine the efficacy and safety of the new injectable implant, Urolastic, in women with SUI after 12-month followup.

## 2. Materials and Methods

This was a clinical, prospective, cohort study that included women with SUI. Inclusion criteria were women >18 years old, with a Stamey grade 1-2 on the Stamey incontinence scale; a patient should not be on anticholinergic treatment unless she has been on stable treatment during the previous month and will continue treatment during the protocol. The bladder capacity should be 300 mL or more and postvoid residual urine of less than 100 mL, and they should have urodynamically proven SUI.

Exclusion criteria were women with urodynamic detrusor overactivity (DO) or predominately urgency incontinence, pelvic organ prolapse (POP), suspicion of neurogenic bladder, long-term indwelling catheter with local fibrotic urethral/bladder neck tissues, pregnancy, or plans of conceiving within 2 years after urethral injection therapy.

The study was approved by the local ethical committee, and all patients signed an informed consent.

### 2.1. Injectable Implant

Urolastic is presented in a prefilled, sterile, dual container of 5 mL (2 syringes × 2.5 mL), supplied with a static mixer that allows for adequate premixing of the syringe content.

### 2.2. Procedure

Patients were put in the lithotomy position. Under local anaesthesia with 1% lidocaine, all patients were treated with Urolastic. An application device was introduced into the urethra. The device allows for periurethral administration of the bulking material. The bulking material was injected through an 18 gauge needle, periurethrally, at the following positions: 2, 6, and 10 o'clock. The urinary bladder was filled up to 200 mL with saline solution at room temperature after which a cough test was performed. Ciprofloxacin 500 mg was prescribed for 5 days after the procedure. The injection procedure was repeated after 6 weeks when indicated.

### 2.3. Assessment of the Outcome

#### 2.3.1. Efficacy

Patients were evaluated at baseline before receiving the final treatment. The follow-up visits were scheduled at 6 weeks, 3 months, and 12 months. The efficacy of the procedure was assessed using the following tools: cough test in supine and standing positions, Stamey scale, and 1 hr pad test. A pad count was calculated at each visit (average over 72 hr before the day of visit), as well as the number of incontinence episodes per 24 hr. Quality of life was assessed with incontinence quality of life (I-QoL) questionnaire.

The primary efficacy endpoint was to determine if the patient will show, and maintain, a decrease in Stamey scale [6] of one or more grades at 3 months and 12 months visits. Grades on Stamey scale were defined as follows: 0 = dry; 1 = urine leakage with vigorous activity; 2 = urine leakage with minor activities; 3 = urine leakage all the time regardless of the activity or position.

The secondary efficacy end points were to detect 50% or more decrease in number of incontinence episodes per day, number of pads used per day, weight of the 1 hr pad test, and 50% or more improvement in I-QoL at 3 months and at 12 months.

#### 2.3.2. Safety

Physical assessment was performed during each visit to detect the frequency and severity of any adverse events related to the injectable implant.

### 2.4. Statistical Analysis

Statistical analysis was done with the Statistica package, version 8.0 (StatSoft Inc., Tulsa, OK, USA). It included testing of normality of data distribution. Wilcoxon Rank test was done to test the difference between outcomes of follow-up visits versus baseline characteristics. The level of significance of the results was set at *P* < 0.05.

The sample size calculation in this study came with a total of 20 evaluable patients with stress urinary incontinence was chosen assuming a true success rate at 3 months of 60%. This sample size has been chosen to get a width of the 95% confidence interval for the estimate of success rate of less than 0.43 (0.60 ± 0.215). To account for an anticipated dropout rate of approximately 20%, a total of 24 patients were selected for the study. Twenty evaluable patients with the most favorable injection result as regarded by the urologist directly after injection therapy entered into this study. Nineteen (19) evaluable patients were available for follow up at 12 months after treatment.

## 3. Results

Twenty women with a mean age of 56 (33–71) years old were included in the study. Three patients had had previous surgical procedure with a midurethral tape for the treatment of their SUI. All patients had a preoperative positive cough test. Thirteen patients (65%) received one session of Urolastic urethral implant, while 7 patients (35%) received a second treatment session. The average volume of Urolastic that was injected in the first session was 2.1 mL (0.47 mL at 2 O'clock, 1.1 mL at 6 O'clock, and 0.52 mL at 10 O'clock positions). An extra average volume of 0.35 mL was injected in the second session.

20 patients completed the follow-up at 6 weeks and at 3 months; 1 patient did not complete the 12 months followup due to loss of contact.

### 3.1. Efficacy Outcomes (Table 1)

The cough test performed after the injection of Urolastic was negative in all patients. The mean Stamey incontinence grade showed a significant decrease from 1.9 at baseline (visit I) to 0.75 at 6 weeks (visit II), 0.2 at 3 months (visit III), and 0.4 at 12 months (visit IV) (*P* < 0.001 for all visits versus baseline).

**Figure 1:**
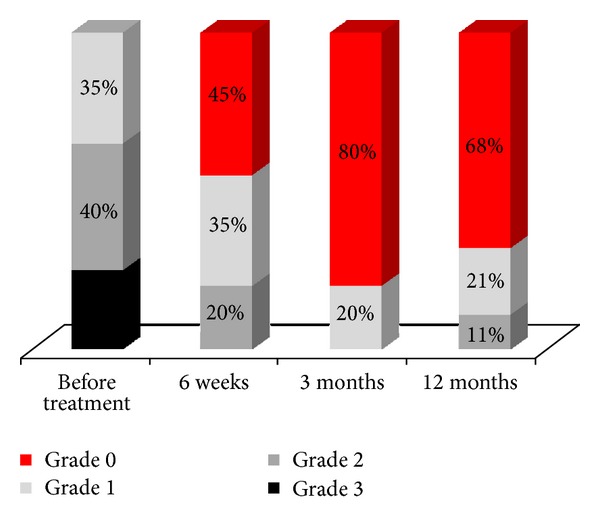
Percentage of particular grades in Stamey scale measured on subsequent visits.

The percentage of patients who were dry at baseline was 0% this percentage increased to be 45% at visit II, 80% at visit III, and 68% at visit IV (Figure 1).

The 1 hr pad test showed a significant decrease in the mean weight of urine loss from 20.2 g at baseline to 5.5 g at visit II, 1.6 g at visit III, and 7.8 g at visit IV (*P* < 0.001 for all visits versus baseline).

The mean number of incontinence episodes per day over 72 hr before the day of visit showed a significant decrease from 6 episodes at baseline to 2.5 episodes at visit II, 1.7 episodes at visit III, and 1.6 episodes at visit IV (*P* < 0.001 for all visits versus baseline).

The mean number of pads used per 72 hr day showed a significant decrease from 17.3 pads at baseline to 8.7 pads at visit II, 5.4 pads at visit III, and 5.6 pads at visit IV (*P* < 0.001 for all visits versus baseline).

The mean I-QoL score showed a significant increase from 51 at baseline to 64 at visit II, 82 at visit III, and 76 at visit IV (*P* < 0.001 for visits III and IV versus baseline; *P* < 0.01 for visit II versus baseline).

### 3.2. Safety Outcome

Six patients (30%) developed complications related to the injection procedure. One patient had a small hematoma after the first injection session, which was resolved spontaneously. Three patients had urinary retention, which was resolved by insertion of urethral catheter for 3 days after which the patients voided spontaneously. Two patients had dyspareunia and vaginal pain, which were resolved after removal of the Urolastic implant at 6 O'clock position. A small incision was made from the vaginal side where the implant could be palpated. Thereafter, the implant was removed using forceps and the wound was closed with a few stitches. Two patients were dry and 1 became incontinent after this procedure.

## 4. Discussion

SUI in women can be either of extrinsic origin due to lack of pelvic floor support to the urethra and bladder neck leading to urethral hypermobility or of intrinsic origin due to weakness in the urethral sphincteric mechanism, that is, intrinsic sphincter deficiency (ISD) [7]. Surgical correction, for example, midurethral slings, can provide a finite cure to most of these conditions. However, these procedures are invasive and are associated with risk of failure, redo, and morbidities. Therefore, many women seek for less invasive therapies with lower rate of complications [3].

Urethral injection therapy with bulking agents has been formerly given to patients with ISD, but the application of these bulking agents has been extended to include patients with SUI due to urethral hypermobility [8, 9]. Urethral bulking agents add bulk to the bladder neck and the proximal urethra; this increases the urethral mucosal coaptation. The resulting increase in the resistance of the bladder neck and proximal urethra can prevent any leakage of urine on exertion or coughing [10].

Many bulking agents have been used in clinical trials and in treatment of SUI, for example, silicon particles, calcium hydroxylapatite, porcine dermis, glutaraldehyde cross-linked bovine collagen, carbon beads, and polyacrylamide hydrogel. However, there is lack and inconsistency of data comparing the outcomes of these agents; therefore, a decision to be made on selecting a bulking agent to treat SUI would be made depending on the availability, safety, ease of use, and surgeons preference [11, 12].

Our study presents the outcome of 12-month followup after initial treatment of 20 women with SUI using Urolastic (Urogyn BV, Nijmegen, The Netherlands). The results show a successful treatment outcome in terms of efficacy and safety of this new bulking urethral implant. The primary efficacy endpoint of our study was successfully achieved as 89% of women who participated in the study showed an improvement of their Stamey scale after 12 months of follow up. The mean Stamey grade was significantly reduced from 1.9 at baseline to 0.4 at 12 months (*P* < 0.001). This result is similar to, or even better than, the 12-month outcome of other studies, which applied Macroplastique bulking agent in the treatment of SUI. ter Meulen et al. [13] reported 10 out of 18 (55%) patients who became dry (Stamey grade 0) after 12 months of initial therapy, Ghoniem et al. [14] reported 57% of patients to be dry after 12 months of initial therapy, land Tamanini et al. [15] reported 73% of their patients to have Stamey grade 0 after 12 months of initial therapy.

The secondary efficacy parameters of our study were shown to be successful too. After 12 months of followup, the 1 hr pad test showed a significant decrease in the mean weight of urine loss (7.8 versus 20.2 at baseline, *P* < 0.001). The mean number of incontinence episodes showed a significant decrease of 1.6 versus 6 at baseline (*P* < 0.001). The mean number of pads showed a significant decrease (5.6 versus 17.3 at baseline, *P* < 0.001). Finally, the I-QoL score sheet showed a substantial improvement 12 months after initial therapy compared to baseline (76 versus 51, *P* < 0.01).

Adverse events commonly seen with various types of urethral bulking agents are development of de novo urgency (24%) and acute urinary retention (17%) [16]. The complications rate in our series seemed to be moderate (30%), but all adverse events were of mild-moderate severity that could easily be treated. Three women experienced urinary retention early after initial injection that was treated by urethral catheter insertion for 3 days and had no effect on the final outcome at 12 months. Ghoniem et al. [17] compared the efficacy of Macroplastique to Contigen in 247 women with the 12-months followup revealing that the Macroplastique was not inferior to Contigen with regard to efficacy (improvement in the Stamey grade), with no serious treatment-related adverse events. The occurrence of postprocedure catheterization wassignificantly higher in patients treated with Macroplastique (43.4% Macroplastique, 24.0% Contigen). Three cases of urethral erosion were observed, two in the Macroplastique group and one in the Contigen group. The major difference between Urolastic and the Macroplastique is that Macroplastique consists of cured silicon particles suspended in a Polyvidone gel base. Particles should be of a distinct size since large particles will occlude the needle and too small particles will migrate. Besides, the gel base is biodegradable leading over time to loss of volume and so loss of effect. Urolastic is a 2-component silicon elastomer, that is, injected while liquid and hardens in situ into a flexible rubberlike plug. It is not degraded but encapsulated by scar tissue. It will maintain its volume and so its effect. It will not migrate.

Minor surgical interference was done to relieve dyspareunia and vaginal pain in 2 patients, which were resolved after removal of the Urolastic implant at the 6 O'clock position.

The overall outcome of 12-month followup of patients in our series revealed that the Urolastic is effective and durable this could be explained by the flexibility of the implant which enables it to adapt itself to the shape of the local environment during injection, thus reducing the chances for migration. Urolastic is also a biocompatible and not biodegradable agent, which gives long-term efficacy.

A limitation of the study is the small sample size a larger study group will justify the statistical and clinical outcome of our small series. Another limitation could be the absence of control arm of the study, which can be explained by the difficulty to do sham procedures in most of the clinical studies.

## 5. Conclusions

Urolastic is an effective and long-standing urethral bulking agent with good and lasting efficacy for one year. It is fairly safe and show moderate adverse events most of which were related to the injection procedure and could be treated with ease.

## Figures and Tables

**Table 1:** Efficacy outcomes versus baseline (visit I) characteristics after 6 weeks (visit II), 3 months (visit III), and 12 months (visit IV).

	Stamey grade	1 hr pad test-weight (g)	Incontinence episodes (24 h)	I-QoL	Number of pads (72 hr)
Baseline (*N* = 20)	1.9 (0.79)	20.2 (24.28)	6.1 (6.70)	50.7 (20.09)	17.3 (14.91)
Visit II (*N* = 20)	0.75 (0.73)^‡^	5.5 (13.98)^‡^	2.5 (2.7)^‡^	64.0 (24.12)^$^	8.7 (8.70)^‡^
Visit III (*N* = 20)	0.2 (0.41)^‡^	1.6 (2.80)^‡^	1.7 (1.9)^‡^	81.9 (21.30)^‡^	5.4 (7.06)^‡^
Visit IV (*N* = 19)	0.4 (0.69)^‡^	7.8 (19.92)^‡^	1.6 (1.77)^‡^	75.5 (20.98)^‡^	5.6 (7.22)^‡^

^‡^ *P* < 0.001 for all visits versus baseline.

^$^ *P* < 0.01 for visit II versus baseline.

Values presented in mean (SD).
